# Recent Advances in the Gastrointestinal Fate of Organic and Inorganic Nanoparticles in Foods

**DOI:** 10.3390/nano12071099

**Published:** 2022-03-27

**Authors:** Hualu Zhou, David Julian McClements

**Affiliations:** Biopolymers and Colloids Laboratory, Department of Food Science, University of Massachusetts, Amherst, MA 01003, USA; hualuzhou@umass.edu

**Keywords:** nanoparticles, gastrointestinal fate, food nanotechnology, digestion, nutraceuticals, bioavailability

## Abstract

Inorganic or organic nanoparticles are often incorporated into foods to enhance their quality, stability, nutrition, or safety. When they pass through the gastrointestinal environment, the properties of these nanoparticles are altered, which impacts their biological effects and potential toxicity. Consequently, there is a need to understand how different kinds of nanoparticles behave within the gastrointestinal tract. In this article, the current understanding of the gastrointestinal fate of nanoparticles in foods is reviewed. Initially, the fundamental physicochemical and structural properties of nanoparticles are discussed, including their compositions, sizes, shapes, and surface chemistries. Then, the impact of food matrix effects and gastrointestinal environments on the fate of ingested nanoparticles is discussed. In particular, the influence of nanoparticle properties on food digestion and nutraceutical bioavailability is highlighted. Finally, future research directions are highlighted that will enable the successful utilization of nanotechnology in foods while also ensuring they are safe.

## 1. Introduction

Nanomaterials are being explored for application in the food industry due to their ability to create new or improved properties in foods and packaging materials [1]. The development of food nanomaterials is an interdisciplinary research effort that includes contributions from the physical, chemical, biological, engineering, and pharmaceutical sciences [1,2].

Nanomaterials have at least one dimension below 100 nm, which includes many kinds of edible fibers, sheets, and particles. However, it should be noted that many food components with larger dimensions (100–1000 nm) are also considered as nanomaterials by some researchers [3,4]. This is because there is typically not a distinct change in the physical, chemical, or biological properties of materials when one of their dimensions falls below 100 nm. Instead, there is typically a more gradual transition in these properties as a material becomes smaller. Some researchers have divided nanomaterials into different categories depending on their attributes. For instance, they have been categorized as either soft or hard nanomaterials [4]. Soft nanomaterials are typically created from organic matter (such as proteins, polysaccharides, and lipids) and tend to be digestible and/or fermentable in the human gastrointestinal tract. In contrast, hard nanomaterials are often constructed from inorganic matter (such as metals or metal salts) and tend to be indigestible and/or non-fermentable. Nanomaterials may also be classified as natural or engineered. Naturally occurring nanomaterials include the casein micelles in milk and the oil bodies in seeds. Engineered nanoparticles are typically designed and synthesized to have compositions and physicochemical properties that lead to specific desirable functional attributes [5]. Both inorganic (such as TiO_2_, Fe_2_O_3_, and Ag) or organic (such as polysaccharides, lipids, and proteins) materials can be used to fabricate engineered nanoparticles suitable for food applications [6].

**Figure 1:**
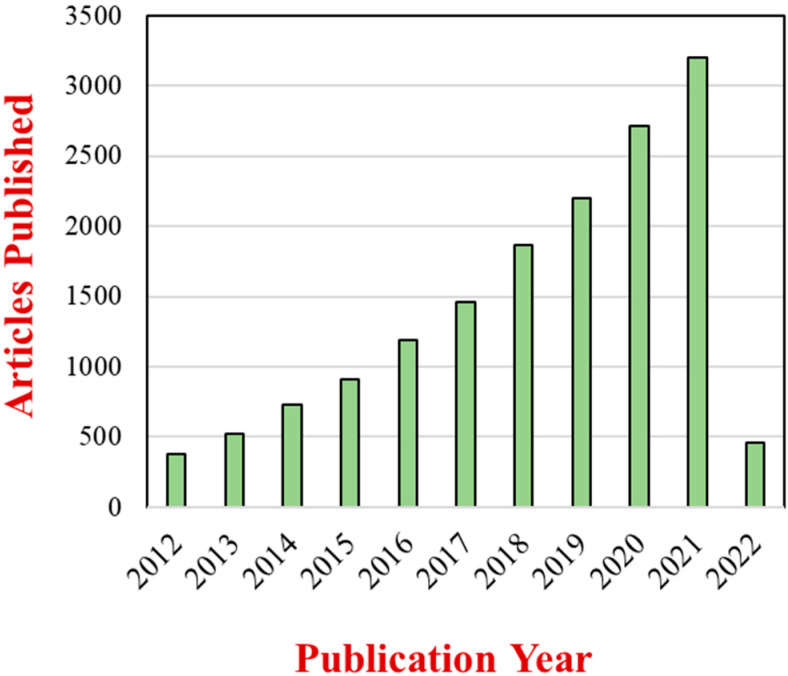
Number of articles with two topic keywords, “nanoparticles” and “foods”, published from 2012 to March 2022 based on a Web of Science Core Collection source.

Nanoparticles are the most common type of nanomaterial currently employed in foods [7]. These nanoparticles come in different compositions, sizes, and shapes, with the most frequently used being spheres, ellipsoids, and fibers. The incorporation of these nanoparticles into foods is often used to improve their optical, flavor, textural, stability, safety, and nutritional properties [8,9]. They have also been also as additives in biodegradable food packaging materials designed to prolong the shelf life of foods [10,11,12]. The extent of the research effort in this area is demonstrated by the continued growth in the number of scientific publications on the applications of nanoparticles in foods (Figure 1).

After ingestion, any nanoparticles present within a food enter the human gastrointestinal tract (GIT) and are exposed to the various conditions that exist in the mouth, esophagus, stomach, small intestine, and colon [8,9]. Exposure to these conditions changes the properties of the nanoparticles, which alters their GIT fate and potential toxicity. Moreover, the nanoparticles themselves may interfere with the different physicochemical and physiological processes occurring within the GIT, such as digestion, transport, metabolism, and absorption. As a result, their presence may alter the digestion and absorption of other components within foods, thereby affecting their pharmacokinetics and bioavailability.

In this review article, we investigate the potential positive and negative effects of incorporating nanoparticles into foods. It is particularly important to consider any potentially undesirable health effects when designing nano-enabled foods [9,13,14]. Previous research suggests that the potential toxicity of nanoparticles depends on their properties, such as their composition, size, shape, and aggregation state [15]. As mentioned earlier, these properties are altered significantly when nanoparticles pass through the GIT, which therefore may impact their toxicity and needs to be considered.

Several authors have reviewed specific aspects of the gastrointestinal fate and potential toxicity of nanoparticles in foods [13,16]. However, there is still a relatively poor understanding of how gastrointestinal conditions affect the physicochemical properties of food-grade nanoparticles. Therefore, in the current article, we focus on the behavior of food-grade inorganic and organic nanoparticles within the human gut, with an emphasis on recent advances in understanding their gastrointestinal fate, especially changes in their biomolecular corona, aggregation state, absorption, and biological effects. Then, this knowledge could be used to improve the functional performance of nano-enabled foods and to avoid undesirable health consequences.

## 2. Intrinsic Properties of Nanoparticles

**Figure 2:**
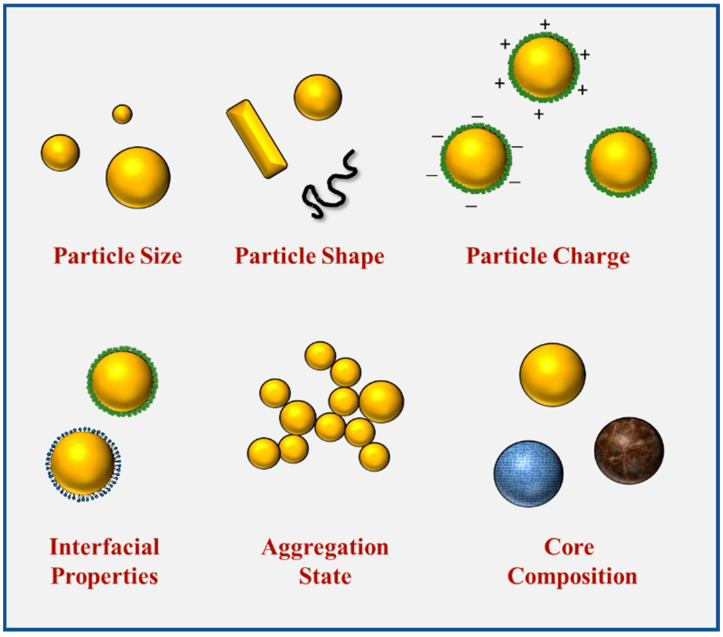
The characteristics of food-grade nanoparticles, including size, shape, composition, aggregation, and interfacial properties.

The intrinsic properties of nanoparticles, such as their size, shape, composition, and interfacial properties, potentially have a major effect on their applications within foods (Figure 2). These properties influence the optical, rheological, flavor, and nutritional attributes of foods and beverages. Moreover, they also affect their behavior within the GIT and therefore impact their digestibility and bioavailability. Therefore, it is important to be able to control and characterize the intrinsic properties of nanoparticles added to foods.

### 2.1. Size and Shape

The dimensions of the nanoparticles used in foods may vary from a few nanometers to a few hundred nanometers depending on their biological origin (for natural nanoparticles) or the ingredients and processing methods used to fabricate them (for engineered nanoparticles). Particle dimensions have a major influence on the physical, chemical, and biological properties of nanoparticles. The optical clarity of nanoparticle dispersions decreases as the particle dimensions decrease, which is important for developing transparent foods and drinks. Moreover, the resistance of nanoparticle dispersions to gravitational separation and aggregation increases as the particle dimensions are reduced, which is useful for increasing the shelf life of foods. After ingestion, the dimensions of the nanoparticles influence their gastrointestinal fate. The rate of digestion of digestible nanoparticles tends to increase as their dimensions decrease, because this increases the surface area exposed to the digestive enzymes in the gastrointestinal fluids [9,13]. Conversely, the ability of indigestible nanoparticles to pass through the pores in the mucus layer and be absorbed by the epithelium cells in the GIT tends to increase as their dimensions decrease [17]. The most common nanoparticles in foods are spherical, but other shapes are possible, such as fibers, ellipsoids, or cuboids [18]. The shape of ingested nanoparticles would also be expected to influence food properties and their biological fate. For instance, the ability of nanoparticles to increase the viscosity of foods tends to increase as their length-to-width ratio increases. Moreover, the ability of nanoparticles to penetrate through the mucus layer is typically easier for spheres than fibers.

### 2.2. Composition

The composition of nanoparticles has a major impact on their behavior in foods as well as their GIT fate. Nanoparticles that only contain inorganic substances may not be digested within the GIT, such as those comprised of TiO_2_. However, some inorganic nanoparticles do dissolve in the acidic gastric environment, such as those consisting of ZnO and Fe_2_O_3_ [19]. Organic nanoparticles are typically fabricated from lipids, proteins, and carbohydrates, which may be either digestible or indigestible depending on the precise nature of the ingredients present [6]. For instance, some lipids are digestible (such as triacylglycerols), whereas others are not (such as mineral oils, essential oils and flavor oils). Similarly, some carbohydrates are digestible (such as starch), whereas others are not (such as cellulose and chitosan). Moreover, the rate of digestion may vary depending on the type of macronutrients used and their organization within the nanoparticles.

### 2.3. Interfacial Properties

The interfacial properties of food-grade nanoparticles, including their composition, charge, polarity, thickness, and chemical reactivity, are also important because they influence their interactions with food matrix and GIT components [20]. The interfacial properties of engineered nanoparticles can be controlled by using different kinds of emulsifiers or other coating materials. It should be stressed that the interfacial properties of nanoparticles change appreciably after they are introduced into foods and as they pass through the GIT [13]. This phenomenon occurs because the original interfacial layers may be digested or displaced from the nanoparticle surfaces and/or because other components in the surroundings can adsorb to their surfaces. As a result, the composition, charge, polarity, thickness, and chemical reactivity of the interfacial layers of the nanoparticles changes, which alters their behavior in foods and the GIT. For instance, interfacial composition is likely to influence the ability of nanoparticles to penetrate through the mucus layer and be absorbed by epithelium cells [13]. Moreover, the relatively large specific surface area of ingested nanoparticles means that they may adsorb components within the gastrointestinal fluids to their surfaces, such as mucin, enzymes, bile salts, mineral ions, and proteins. As a result, the presence of the nanoparticles could interfere with normal digestion processes.

## 3. Types of Food Nanoparticles

### 3.1. Organic Nanoparticles

**Figure 3:**
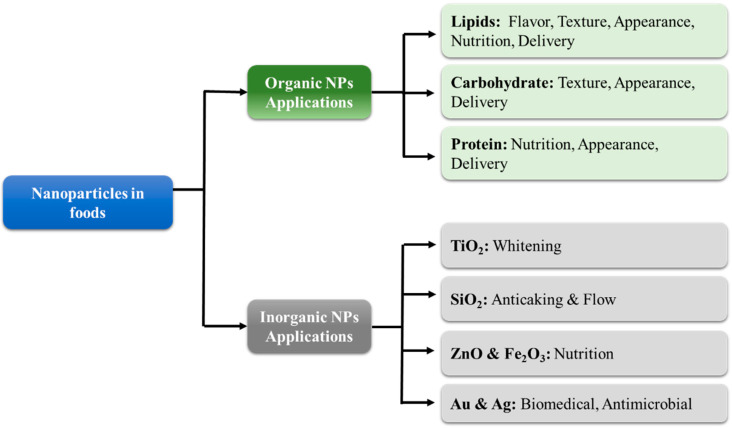
The categories and applications of organic and inorganic nanoparticles in foods.

Organic nanoparticles are typically fabricated from lipids, proteins, and/or carbohydrates (Figure 3). These nanoparticles are usually incorporated into foods to provide desirable optical, textural, flavor, or nutritional attributes [18]. In general, the safety of organic nanoparticles is of less concern than that of inorganic ones because they are usually fully digested. For this reason, food scientists are typically more focused on their fabrication and utilization as functional ingredients in foods [9]. Nevertheless, there may be some potential toxicity concerns with digestible nanoparticles, such as their ability to greatly increase the bioavailability of encapsulated or co-ingested hydrophobic substances. In most cases, this is advantageous, but in some cases, it may lead to problems. For instance, they could increase the absorption of undesirable hydrophobic substances in foods, such as pesticides [21]. Alternatively, they could increase the bioavailability of food components into a region where they exhibit some toxicity.

#### 3.1.1. Carbohydrate Nanoparticles

The main structural components used to fabricate carbohydrate-based nanoparticles are polysaccharides. This type of nanoparticle can be fabricated using various approaches. For example, carbohydrate nanoparticles can be assembled by bottom–up methods that are based on the self-assembly of polysaccharides under appropriate environmental conditions, e.g., pectin, alginate, carrageenan, agar, starch, and chitosan [22,23,24]. Typically, solution or environmental conditions, such as solvent quality, pH, ionic strength, enzyme activity, or temperature, are changed to promote the formation of physical or chemical crosslinks between the polysaccharide molecules. Conversely, nanocrystals or nanofibers can be obtained using top–down methods that utilize the controlled disintegration of bulk polysaccharide materials, such as acid or alkaline treatment of cellulose or chitin [25,26]. Carbohydrate nanoparticles are often used as carriers for nutraceuticals, vitamins, or other bioactive agents, but they may also be used as texture modifiers, film formers, lightening agents, and UV-light blockers. After oral intake, the gastrointestinal fate of carbohydrate nanoparticles is strongly related to their digestibility. Nanoparticles assembled from digestible polysaccharides, such as some types of starch, are hydrolyzed in the mouth and small intestine by amylases. Conversely, nanoparticles fabricated from indigestible polysaccharides, such as cellulose, chitin, or gums, are not hydrolyzed until they reach the colon, where they may be fermented by colonic bacteria. These characteristics also influence the pharmacokinetics and bioavailability of other bioactive components encapsulated within carbohydrate nanoparticles [27]. For instance, if a bioactive component needs to be delivered to the mouth, then it can be encapsulated within digestible starch nanoparticles. If it needs to be delivered into the small intestine, then it can be encapsulated inside protein nanoparticles. Finally, if it needs to be delivered into the colon, then it can be encapsulated within dietary fiber-based nanoparticles.

#### 3.1.2. Protein Nanoparticles

Protein nanoparticles have also been explored for many years for their potential utilization as functional ingredients in foods and beverages [28]. Casein micelles are natural nanoparticles found in milk that are assembled from protein, calcium, and phosphate, which are held together by physical forces such as electrostatic and hydrophobic attraction. Engineered protein nanoparticles have also being widely explored for their utilization as carrier systems, texture modifiers, fat replacers, and lightening agents [28]. A number of different preparation methods have been developed, with the most suitable one depending on the nature of the proteins. For example, protein nanoparticles can be formed from hydrophobic proteins, such as zein or gliadin, using an antisolvent precipitation method [29]. In this method, the hydrophobic proteins are first dissolved within a good solvent, such as a concentrated ethanol solution. Then, this solution is added to a bad solvent, such as water, which leads to the spontaneous formation of protein nanoparticles because the hydrophobic protein molecules do not want to be in contact with water and so interact with each other [18]. The hydrophobic interior of this kind of nanoparticle is particularly suitable for the encapsulation of lipophilic bioactives, such as oil-soluble vitamins and nutraceuticals [30]. Protein nanoparticles can also be assembled from hydrophilic proteins by heating them to a temperature above their thermal denaturation temperature, which causes them to unfold and expose hydrophobic groups. As a result, the protein molecules self-assemble into nanoparticles to reduce the number of hydrophobic groups exposed to water. It is more difficult to entrap bioactives into nanoparticles assembled from hydrophilic proteins using this method, but the nanoparticles can be used as texture modifiers, lightening agents, or fat replacers. The charge on bare protein nanoparticles tends to move from positive to negative as the pH is raised from below to above their isoelectric points. Around their isoelectric point, they tend to aggregate because the weak electrostatic repulsion between them is not strong enough to overcome the van der Waals or hydrophobic attraction. For this reason, protein nanoparticles are often coated with surfactants or charged biopolymers to increase the repulsive forces or decrease the attractive forces acting between them, thereby improving their resistance to aggregation.

The GIT fate of protein nanoparticles depends on their size, composition, and surface characteristics. They are typically digested within the stomach and small intestine due to the presence of gastric (pepsin) and pancreatic (trypsin and chymotrypsin) proteases. However, the rate of digestion may depend on the type of proteins used, their conformation, and any coating materials used. Consequently, it is possible to control the retention and release of bioactives inside protein nanoparticles.

#### 3.1.3. Lipid Nanoparticles

Lipid nanoparticles have been the focus of many studies on the application of nanotechnology within foods and beverages and are already widely utilized in some food and beverage products. This kind of nanoparticle is formed during the homogenization of milk and beverages, including many soft drinks. They may also be produced using various other high-energy (mechanical) and low-energy (physicochemical) methods [31]. For instance, they can be produced using sonicators or microfluidizers (high-energy) or using spontaneous emulsification or phase inversion temperature methods (low-energy). Lipid nanoparticles are often utilized as carriers for lipophilic nutraceuticals and oil-soluble vitamins, which can help solve challenges associated with their poor water solubility, chemical stability, and bioavailability characteristics [18]. Numerous kinds of lipid nanoparticles have been developed as carriers, including micelles, nanoliposomes, nanoemulsions, and solid lipid nanoparticles [2]. Polar lipids such as phospholipids that contain a hydrophilic “head” and two hydrophobic “tails” can form liposomes, whereas non-polar lipids such as triacylglycerols can be used to form the core of oil droplets or solid fat particles.

In the GIT, digestible lipid nanoparticles, such as those fabricated from triacylglycerols, are digested by lipase in the stomach and small intestine, whereas indigestible ones, such as those fabricated from essential, flavor, or mineral oils, are not. Lipid nanoparticles have relatively large specific surface areas, which means that lipase can rapidly adsorb to their surfaces, leading to rapid lipid digestion and bioactive release [32]. However, the emulsifier used to coat the lipid nanoparticles also plays an important role in determining their GIT fat because it can alter their aggregation state in the GIT or inhibit the adsorption of bile salts or lipase [33,34]. Consequently, it is important to select an appropriate emulsifier type and oil phase for the specific application.

#### 3.1.4. Composite Nanoparticles

Carbohydrates, proteins, and lipids can be used in combination to create composite nanoparticles [35]. Many proteins and carbohydrates have hydrophilic and lipophilic areas on their surfaces and so can act as emulsifiers that promote the formation and stabilization of lipid nanoparticles [36]. Polysaccharides and proteins that have opposite charges can be made to assemble into composite nanoparticles via electrostatic attraction. Composite nanoparticles have great potential within the food industry because their functional properties can be tailored for particular applications.

### 3.2. Inorganic Nanoparticles

Several kinds of inorganic nanoparticles can also be used in foods for their functional attributes (Figure 3), such as their ability to alter the appearance, texture, stability, or nutritional profile of foods or packaging materials. However, as mentioned earlier, there is often more concern about the potential toxicity of inorganic nanoparticles in foods than organic ones because they are not digested in the GIT but may still be absorbed by the human body [37]. In this section, a brief overview of some of the inorganic nanoparticles that are commonly used in foods or food packaging materials is given.

#### 3.2.1. Titanium Dioxide (TiO_2_) Nanoparticles

Titanium dioxide is commonly used in food products as a brightening or whitening agent because the crystalline material is white and has a high refractive index so that TiO_2_ particles scatter light strongly. For this reason, it has been widely in foods to improve their visual appearance, most notably candies, chewing gums, bakery goods, and milk powders. The powdered TiO_2_ used as an additive in the food industry (E171) contains a broad range of differently sized particles, with an appreciable fraction falling into the nano-range. Indeed, analysis of a commercial TiO_2_ food additive found that more than 36% of the particles had diameters below 100 nm [38]. These nanoparticles are likely to behave differently in the human gut than the larger particles because of their small dimensions and high surface areas, which has raised some health concerns [39,40]. Indeed, the French government recently banned the use of this kind of nanoparticles in foods due to these concerns [41]. However, it should be noted that the potential toxicity exhibited by TiO_2_ nanoparticles in cell culture and animal feeding experiments varies considerably from study to study. Some studies suggest that TiO_2_ nanoparticles alter the gut microbiota or accumulate in the tissues of mammals and other vertebrates with a low elimination rate, whereas others indicated low toxicity and accumulation [42,43,44]. Part of the variations in findings between studies may be because the impact of food matrix effects and GIT conditions on the gastrointestinal fate of TiO_2_ nanoparticles is often ignored as well as due to differences in the nature of the TiO_2_ nanoparticles used.

#### 3.2.2. Silicon Dioxide (SiO_2_) Nanoparticles

Powdered silicon dioxide is commonly used as an additive in the food industry because it is capable of strongly absorbing water, thereby acting as an anticaking agent [45]. For this reason, it is often incorporated into powdered foods such as salts, dried milk, and icing sugar to prevent clumping and to enhance their flow properties. Most of the particles in commercial food-grade SiO_2_ additives (E551) fall between 100 and 1000 nm in diameter, but an appreciable proportion may fall into the nano-range [46]. Some cell culture studies have indicated that high levels of SiO_2_ nanoparticles caused cytotoxic and genotoxic effects [47]. However, another study reported no accumulation or toxicity of this type of nanoparticle when it was fed to rats [48]. Again, there appear to be large variations in the potential toxicity of this kind of inorganic nanoparticle reported in different studies. This apparent discrepancy may be because of differences in the nature of the SiO_2_ additives tested (such as dose, size, aggregation state, and surface characteristics), as well as differences in food matrix and GIT effects.

#### 3.2.3. Zinc Oxide (ZnO) and Iron Oxide (Fe_2_O_3_) Nanoparticles

Zinc and iron are important micronutrients that are often lacking from the human diet, and so foods are often fortified with bioavailable forms of these essential minerals [49]. For this reason, powdered ZnO and Fe_2_O_3_ additives are sometimes used as a source of these minerals in functional foods and nutritional supplements. As with other inorganic additives, these powders are engineered particles, which likely contain a range of different particle sizes with some falling within the nano-scale range [50]. These additives may also be incorporated into food packaging materials because of their strong antimicrobial activity. ZnO nanoparticles can penetrate through microbial cell walls and generate reactive oxygen species (ROS) inside the cells, thereby damaging critical cellular components and interfering with key biochemical pathways, ultimately leading to cytotoxicity [51]. Fe_2_O_3_ nanoparticles have also been reported to generate ROS and promote oxidative stress in human lymphocytes [52]. There are also concerns that these nanoparticles could penetrate into human cells after ingestion and have similar effects [53]. However, the extent of these effects depends on the dose used. It has been estimated that people typically consume around 0.45 mg/day of iron oxide, but the amount taken from dietary supplements may range from 10 to 32 mg/day [54]. Nevertheless, a rat feeding study reported no appreciable tissue accumulation or toxicity for this type of nanoparticle, even when it was administered by relatively high doses (250–10,000 mg/kg body weight) [55]. Unlike TiO_2_ and SiO_2_ particles, ZnO and Fe_2_O_3_ particles may be dissolved in the acidic gastric fluids, thereby altering their gastrointestinal fate [56].

#### 3.2.4. Gold and Silver Nanoparticles

Colloidal forms of gold or silver nanoparticles have been used in a diverse range of applications within foods and biomedicines [57,58]. The applications of golden nanoparticles in the biomedical field have been reviewed in detail in several recent articles, including drug delivery, bioimaging, and cancer therapy [59,60,61]. They have also been used to formulate food packaging materials, which may also lead to oral exposure, e.g., due to the migration of silver nanoparticles from packages into foods [62]. In principle, any silver nanoparticles present in packaging materials that are in contact with foods may diffuse into the foods themselves and therefore be ingested. However, the extent of this effect depends on the nanoparticles, packaging materials, and storage conditions used. Nevertheless, it has been estimated that the amount of silver consumed by human adults (20 to 80 μg/day) is relatively low, and only a fraction of this is actually silver nanoparticles [14]. Some studies have reported that a small fraction (<1%) of ingested silver nanoparticles can accumulate in tissues but that most of them are excreted in the feces or urine [63]. Thus, the potential adverse effects of silver nanoparticles still remain inconclusive, and more studies are needed, especially on their long-term chronic toxicity. It should be noted that silver can undergo chemical reactions in foods and the GIT that could alter its gastrointestinal fate. For instance, silver may be oxidized to silver oxide in air, which can dissolve under acidic conditions, or silver ions can precipitate when they come into contact with chloride ions, thereby leading to the possible formation of new nanoparticles [64,65].

## 4. Gastrointestinal Fate of Food-Grade Nanoparticles

### 4.1. Gastrointestinal Conditions

**Figure 4:**
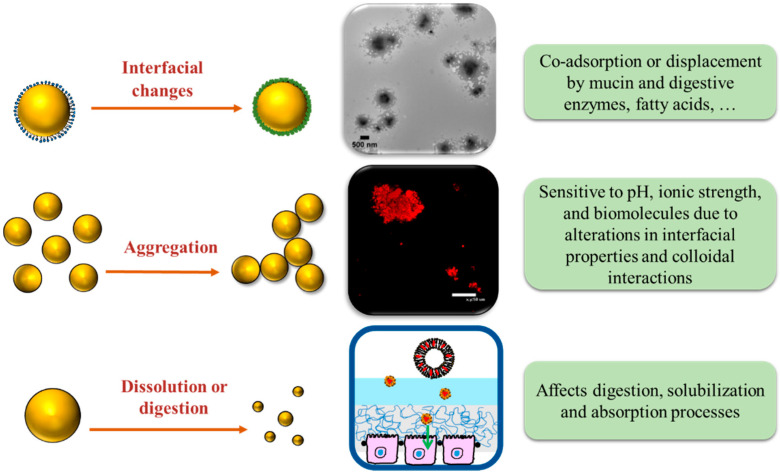
Schematic representation of changes in the properties of nanoparticles within the GIT after ingestion. Transmission electron microscopy (TEM) and confocal laser scanning microscopy (CLSM) images are adopted from our previous studies [68,69]. The scale bars are 0.5 and 50 μm, respectively.

The human gut is designed to protect the body from harmful substances and to effectively break down and absorb nutrients [66]. It consists of a continuous integrated system consisting of the mouth, esophagus, stomach, small intestine, and large intestine. Each region has a characteristic range of transit times, mechanical forces, and chemical/biochemical conditions, such as pH, mineral composition, digestive enzymes, mucin, bile salts, and other constituents [67]. The unique environments within the different regions of the human gut play a critical role in determining the gastrointestinal fate of ingested nanoparticles because they may alter particle characteristics, such as size, aggregation state, interfacial composition, and charge (Figure 4).

Many of the mechanistic insights into the gastrointestinal fate of nanoparticles have come from the utilization of simulated in vitro digestion models [70]. Recently, the standardized INFOGEST model has been widely adopted and used for this purpose [67]. This model simulates the main features of the upper human GIT, including temperatures, transit times, mechanical forces, and chemical/biochemical compositions. A detailed description of these factors is given in the original paper. Here, we provide a summary of the main features of this model: *Mouth*: The food sample to be tested is mixed with simulated salivary fluids (SSFs) containing minerals, mucin, and amylase for a few minutes (pH 7, 310.15 K). *Stomach*: The sample from the mouth phase is mixed with simulated gastric fluids (SGFs) that contain minerals, pepsin, and gastric lipase and incubated for 2 h (pH 3, 310.15 K). *Small intestine*: The sample from the stomach is mixed with simulated intestinal fluids (SIFs) containing minerals, bile salts, and pancreatin (lipase, chymotrypsin, trypsin, amylase) and incubated for 2 h (pH 7, 310.15 K). During this period, the extent of macronutrient digestion can be measured by periodically selecting samples and measuring the extent of lipid, protein, or starch hydrolysis.

In cases where researchers are interested in understanding the behavior of nanoparticles under large intestine conditions, it is also possible to use in vitro colonic models [71]. These models typically include a mixture of different gut microbes like those found in humans, which can secrete enzymes and ferment any non-digested macronutrients and dietary fibers.

In general, ingested nanoparticles experience a complex set of environmental conditions as they pass through the different segments of the human gut, including variations in pH, ionic composition, enzyme activity, bile salt and phospholipid levels, and ingredient interactions. As a result, there may be appreciable changes in their structural and surface properties in different GIT segments. Moreover, the presence of the nanoparticles may interfere with the different physicochemical and biochemical events normally occurring in these regions, thereby altering macronutrient digestion or bioactive bioavailability (Table 1). Therefore, understanding these processes can provide important insights into the design of nanoparticle-based delivery systems that are more effective and safer.

### 4.2. Formation of Biomolecular Coronas

After fabrication, organic or inorganic nanoparticles typically have a specific surface chemistry that is determined by the material they are fabricated from or the kinds of surface-active molecules used to stabilize them. Once they are incorporated into food matrices, which typically contain a range of different surface active and/or charged substances, their surface chemistry may change. Similarly, after ingestion, the composition and structure of nanoparticle interfaces may change appreciably due to the digestion or displacement of the original surface molecules, or as a result of adsorption of surface-active materials in the gastrointestinal fluids, such as mucin, proteins, peptides, bile salts, phospholipids, or mineral ions [8,9]. These surface-active substances may be located between or on top of the original surface molecules. The formation of a biomolecular corona around the nanoparticles impacts their physicochemical properties and gastrointestinal fate [80]. For instance, the formation of a biomolecular corona can change their surface chemistry and aggregation state, which may alter the ability of enzymes to be adsorbed on the nanoparticles. Moreover, they may affect the ability of nanoparticles to penetrate through biological barriers, such as the mucus layer or epithelium cells, by altering their aggregation state and therefore effective dimensions. The impact of some important food matrix and gastrointestinal effects on the formation of biomolecular coronas around nanoparticles is discussed in the remainder of this section.

Studies have shown that α-amylase can adsorb to the surfaces of chitin nanowhiskers (CNWs) under simulated oral conditions, which was attributed to hydrophobic interactions between the enzymes and polysaccharide nanofibers [81]. Some of the α-amylase was tightly bound to the CNWs and formed a “hard” corona, whereas the rest was loosely bound and formed a “soft” corona. The presence of the biomolecular corona was shown to alter the aggregation stability of the CNWs, which would be expected to change their behavior in the GIT. Mucin has been shown to adsorb to the surfaces of food-grade TiO_2_ nanoparticles under simulated oral conditions and form a biomolecular corona [68], which was again mainly attributed to hydrophobic interactions. Other studies have indicated that lipophilic or amphiphilic biomolecules, such as lipids and proteins, can also adsorb to the surfaces of TiO_2_ nanoparticles [72]. In general, it would be expected that a wide variety of surface-active or charged molecules in foods or saliva could bind to the surfaces of TiO_2_ nanoparticles within the mouth, but future studies are required to confirm this.

The biomolecular corona of nanoparticles may be further altered after they enter the gastric chamber due to the presence of various enzymes (such as lipases and proteases), digestion products (such as free fatty acids and peptides), and GIT secretions (such as mucin) that may be surface active or charged. For instance, it has been reported that pepsin can form a protein corona on silver nanoparticles under simulated gastric conditions [65].

Organic nanoparticles may also experience changes in their biomolecular corona when exposed to gastrointestinal conditions. For example, mucin, digestive enzymes, and free fatty acids can adsorb to the surfaces of the lipid droplets in nanoemulsions by an amount that depends on the nature of the emulsifiers used [72]. Studies have also reported that digestive enzymes can form a coating around cationic polymeric nanoparticles, which influenced their uptake by Caco-2 cells [82]. Other studies have shown that cellulose nanocrystals may become trapped in the intestinal mucus layer and therefore fail to reach the underlying epithelium cells [22,23].

In summary, there is strong evidence that a biomolecular corona forms around both organic and inorganic nanoparticles under GIT conditions, and that this can affect the fate of the nanoparticles. However, further systematic research is required with different kinds of nanoparticles and gastrointestinal constituents to better understand this process. In addition, more research is needed to assess whether the formation of a biomolecular corona impacts the cellular toxicity of ingested nanoparticles [72].

### 4.3. Nanoparticle Aggregation

The aggregation of nanoparticles within the GIT system will affect their digestion and absorption. The aggregation state of nanoparticles may change considerably within the human gut due to changes in factors such as pH, mineral composition, ingredient interactions, and surface chemistry. In particular, pH is known to have a major impact because this can alter the electrical charge on nanoparticle surfaces, which influences their tendency to interact with each other or with other GIT components.

In general, charged nanoparticles have a greater tendency to aggregate at high ionic strengths (due to electrostatic screening) or at pH values where the surface potential is relatively low (low surface charge density) [83]. Studies have shown that the adsorption of pepsin onto the surfaces of silver nanoparticles can promote their aggregation, which can be attributed to bridging flocculation and charge neutralization effects [84]. In another study, it was shown that silver nanoparticles aggregated when exposed to gastric conditions but then became non-aggregated when exposed to small intestine conditions [74], which was again mainly attributed to changes in electrostatic interactions. For the organic nanoparticles, lipid nanoparticles stabilized by an ionic surfactant have been shown to aggregate when exposed to high salt concentrations due to electrostatic screening effects [85].

In addition to surface charge, the aggregation stability of organic nanoparticles may also change appreciably depending on their interfacial composition. For example, many protein-stabilized nanoparticles tend to aggregate in different regions of GIT system because of changes in their surface structure or charge. In particular, they may aggregate under gastric conditions due to pepsin digestion of the interfacial layer or due to bridging flocculation caused by the adsorption of anionic mucin onto the cationic protein-coated nanoparticles [33,86]. In contrast, nanoparticle aggregation is not observed under gastric conditions when they are coated by small molecule surfactants, such as Tween 20, Tween 80, or quillaja saponin, since these emulsifiers are resistant to gastric digestion and generate strong steric repulsive forces [33,87]. Therefore, the tendency for nanoparticles to aggregate under GIT conditions depends on their surface properties, such as charge, thickness, and resistance to digestion. It should be noted that aggregates formed in the stomach may break down in the small intestine because of the change in pH and enzyme activities, which would influence the digestion and absorption of nanoparticles.

### 4.4. Impact on Digestive Enzyme Activity

The presence of nanoparticles in the GIT may also affect the activity of digestive enzymes, such as amylases, lipases, and proteases, thereby interfering with macronutrient digestion. There are several potential mechanisms whereby ingested nanoparticles could either increase or decrease lipid digestions. For digestible nanoparticles, the rate of digestion tends to increase as the particle size decreases, because this leads to a bigger surface area for the digestive enzymes to adsorb. For indigestible nanoparticles, the large specific surface area of the particles means that digestive enzymes and other gastrointestinal constituents in their surroundings can adsorb to their surfaces. As a result, they may no longer be able to participate in normal GIT processes. For instance, the binding of digestive enzymes to nanoparticle surfaces may reduce their ability to hydrolyze macronutrients.

Several representative studies that have focused on the impact of nanoparticle characteristics on macronutrient digestion are highlighted here, with an emphasis on lipid digestion, since most studies have been carried out in this area. Numerous researchers have shown that the rate of lipid digestion increases as the oil droplet size decreases, which is mainly attributed to an increase in the surface area of lipids exposed to lipase [75,88]. Studies have also shown that the nature of the emulsifier used to coat the lipid droplets is important, as this impacts the aggregation state of the droplets as well as the ability of lipase to adsorb [34,89].

The incorporation of additives into lipid nanoparticle dispersions can also affect their digestion under GIT conditions. For instance, the addition of dietary fibers (such as pectin, methyl cellulose, and chitosan) has been shown to alter the rate and extent of in vitro lipid digestion in nanoemulsions, which was attributed to their ability to promote droplet flocculation and bind with gastrointestinal species such as bile salts, fatty acids, calcium, and enzymes [90]. The addition of nanochitin has also been reported to suppress lipid digestion in nanoemulsions for similar reasons [69,91].

The potential impact of inorganic nanoparticles on lipid digestion has also been examined [73]. For example, it was observed that incorporating TiO_2_ nanoparticles (either 18 or 167 nm) only caused a small reduction in lipid digestion in nanoemulsions, which was attributed to some adsorption of the lipase to the inorganic nanoparticle surfaces [73]. Other researchers have also reported that TiO_2_ nanoparticles can inhibit lipase activity, which can be attributed to a similar mechanism [92]. In addition, the inorganic nanoparticles may also bind free fatty acids to their surfaces, thereby altering the formation of mixed micelles, which could also reduce the ability of the micelles to solubilize bioactive agents [93].

Nanoparticles can also affect the digestion of proteins. The digestibility of casein molecules depends on whether they are present in casein micelles (natural nanoparticles) or at the surfaces of lipid droplets, which is probably because their location alters the accessibility of the peptide bonds to proteases [88,94]. The presence of cationic TiO_2_ nanoparticles has been reported to reduce the digestibility of anionic casein molecules by forming nanoparticle–protein complexes, which may shield the peptide bonds from proteases [95]. It should be noted that many researchers do not explicitly consider the impacts of food matrix and gastrointestinal effects on the behavior of ingested nanoparticles. In future studies, it will be important to take these effects into account to obtain a more accurate understanding of the potential behavior of nanoparticle in the human gut.

### 4.5. Impact on Bioactive Bioavailability

There are a number of bioactive components in foods that are essential for human health and well-being or that may help inhibit the onset of certain diseases, such as vitamins, minerals, and nutraceuticals [96]. However, many of these components have a relatively low bioavailability because they have poor solubility or stability in gastrointestinal fluids, or they have low absorption [97]. For this reason, nanoparticle-based carriers designed to encapsulate, protect, and release these bioactive components are being developed to increase their oral bioavailability [98]. Most of these carriers are assembled from organic food-grade materials, such as lipids, proteins, carbohydrates, and emulsifiers [16,99].

The oral bioavailability of a bioactive substance can be defined as the amount reaching the systemic circulation in an active form divided by the total amount ingested. The overall bioavailability can be divided into three different contributions: $F=F_{B}\times F_{A}\times F_{M}$. Here, *F*_B_ is the fraction in a bioaccessible form within the small intestine, *F*_A_ is the fraction absorbed into the systemic circulation, and *F*_M_ is the fraction in an active form after metabolism or chemical transformation [100]. Which of these factors acts as a rate-limiting step depends on the nature of the bioactive agent. For instance, for strongly hydrophobic vitamins and nutraceuticals, the rate-limiting step is often their bioaccessibility, because their low solubility in the intestinal fluids limits the amount that is available for absorption. The composition, structure, and physicochemical properties of nanoparticle-based carriers plays a major role in determining the bioavailability of bioactive agents [101]. The biological fate of drug nanocarriers and the impacts of nanoparticle properties on the drug bioavailability have recently been reviewed, which may be useful for understanding the gastrointestinal fate of vitamins and nutraceuticals also [102,103]. Therefore, in the remainder of this section, we focus on the major factors that affect the bioavailability of bioactive agents, especially lipophilic vitamins and nutraceuticals.

Some of the most important characteristics of nanoparticle-based carriers that influence the bioavailability of bioactive agents are the particle size, oil phase composition, and interfacial chemistry. In addition, the molecular characteristics of the bioactive agents themselves are important, particularly their dimensions, polarity, and chemical reactivity.

Studies of the impact of droplet size on the bioaccessibility of carotenoids trapped in the lipid carriers in nanoemulsions or emulsions have found that β-carotene bioaccessibility increased as the initial particle dimensions decreased: d_43_ ≈ 23, 0.4, and 0.2 μm [104]. This effect can be attributed to two effects: (i) the fraction of non-digested lipids decreases with decreasing particle size, so there was a greater release of the carotenoids; and (ii) the amount of mixed micelles formed increases with the decreasing particle size, so there was a greater solubilization of the released carotenoids.

The relative molecular dimensions of bioactive agents and the hydrophobic cores of mixed micelles (which depends on carrier lipid type) also play an important role in determining bioaccessibility. For large hydrophobic bioactive agents, such as vitamin E, β-carotene, and Co-enzyme Q10, it is important to use long-chain triglycerides as carrier lipids since they form mixed micelles with hydrophobic domains that are large enough to solubilize them. In contrast, if short or medium-chain triglycerides are used as carrier lipids, the hydrophobic interiors of the mixed micelles formed are not large enough to incorporate the bioactive molecules [105]. For smaller bioactive agents, such as curcumin or 5-demethylnobiletin, it is possible to solubilize them in carrier lipids consisting of either medium or long-chain triglycerides, since they can easily fit into the mixed micelles formed [106].

The interfacial chemistry of nanocarriers also influences the bioaccessibility of encapsulated bioactive agents by altering their aggregation state under GIT conditions or by altering the ability of lipase to adsorb to the nanocarrier surface. For example, the hydrophilic/lipophilic balance (HLB number) of surfactants was shown to influence the bioaccessibility of hydrophobic bioactive agents in nanoemulsions [106]. A recent study in our laboratory showed that the bioaccessibility of β-carotene in nanoemulsions depended on emulsifier type: lysolecithin (25%) < gum arabic (51%) < caseinate (55%) < quillaja saponin (56%) < Tween 20 (62%). This effect was attributed to the impact of emulsifier type on droplet flocculation, lipid digestion, β-carotene release, and β-carotene protection from chemical degradation. Overall, these experiments highlight the importance of designing nanoparticle-based carriers carefully to maximize the bioavailability of the encapsulated components.

**Figure 5:**
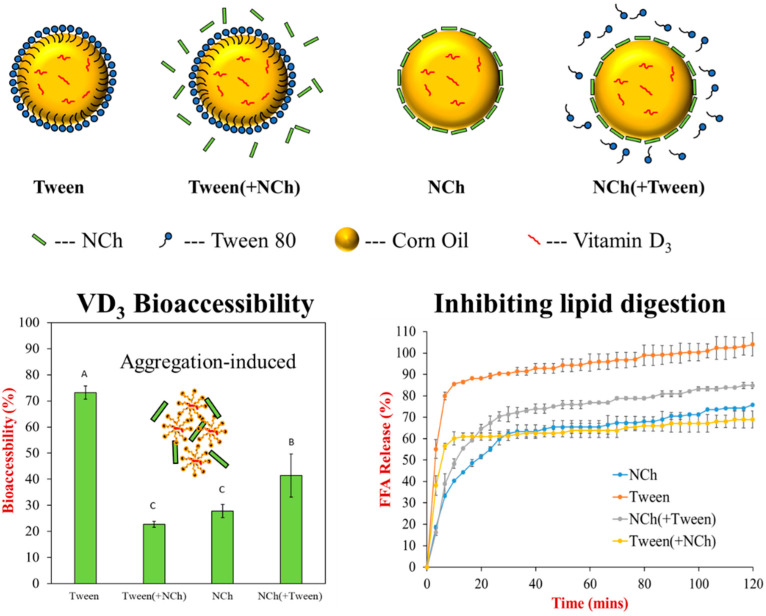
Impact of nanochitin (free or adsorbed) on lipid digestion and vitamin D_3_ bioaccessibility in oil-in-water emulsions. Adapted from reference [69].

We now consider the situation where non-digestible organic or inorganic nanoparticles are added to another system containing bioactive agents, typically hydrophobic bioactives loaded into lipid droplets. The addition of these other nanoparticles can affect the bioavailability of the bioactive molecules in several ways [69,91]. First, these nanoparticles could adsorb to the surface of the lipid droplets and form a shell that inhibits the ability of the lipase molecules to adsorb, thereby inhibiting lipid digestion and reducing bioaccessibility (Figure 5). Second, the presence of these nanoparticles can promote the aggregation of the lipid droplets, again reducing digestion and bioaccessibility. Third, the nanoparticles could bind to gastrointestinal components, such as bile salts or lipase, thereby reducing their ability to interact with the lipid droplets. Indeed, it has been reported that nanochitin can reduce the bioaccessibility of vitamin D_3_ and β-carotene in emulsions through these mechanisms [69,91].

Lipid digestion and nutraceutical bioaccessibility can be also affected by the presence of mineral ions in foods. High levels of calcium ions have been reported to increase lipid digestion but decrease β-carotene bioaccessibility in nanoemulsions, which was attributed to the ability of the cationic calcium ions to precipitate the anionic β-carotene-loaded mixed micelles through an electrostatic interaction [107]. High levels of calcium have also been shown to reduce vitamin D bioaccessibility in nanoemulsions through the same mechanism [108]. These studies suggest that the presence of mineral ions can alter the gastrointestinal fate of food-grade nanoparticles.

## 5. Conclusions and Future Trends

The application of nanomaterials in foods has been the focus of a great deal of research over the past two decades. This research has identified several potentially promising applications for nanotechnology in the food industry, including using nanoparticles to improve the quality, shelf life, safety, and nutritional profile of foods as well as to enhance the functional performance of packaging materials. However, the design of safe and efficacious nano-enabled products depends on understanding their gastrointestinal fate after ingestion. This article has reviewed the properties and applications of different kinds of organic and inorganic food-grade nanoparticles as well as the physicochemical principles underlying their fate within the human gut. In particular, the importance of the food matrix and gastrointestinal conditions on the properties of nanoparticles, such as their size, aggregation state, and surface chemistry has been highlighted, since these factors are known to affect their GIT fate. In future studies, it would be advantageous to carry out more in vivo studies of the gastrointestinal fate of nanoparticles, especially using animal and human feeding trials. Moreover, it will be important to correlate the results from in vitro digestion models (such as the widely used INFOGEST method) and in vivo studies. Then, this knowledge could be used to establish the validity of the current in vitro models as well as to assess the behavior of ingested nanoparticles under more realistic conditions.

## Figures and Tables

**Table 1:** Selected examples of studies on the gastrointestinal fate of different kinds of inorganic and organic nanoparticles (NPs).

Systems	In Vitro/In Vivo Study	Gastrointestinal Fate	Reference
TiO_2_ NPs (E171) added in simulated food models	In vitro GIT model	Biomolecules adsorb to nanoparticle surfaces and form protein- or lipid-based coronas. The size had little impact on lipid droplet aggregation or digestion of food emulsions.	[72,73]
Fe and Fe/Zn NPs	Rat feeding study	Increased iron bioavailability.	[55]
Ag NPs	In vitro GIT model	Formed protein coronas and aggregate due to a pH decrease and digestive enzymes.	[74]
Cellulose or chitin nanocrystals in Pickering emulsions	In vitro GIT model	Cellulose nanocrystals trapped in mucus layer and so failed to reach epithelium cells. Chitin nanocrystals induce fat droplet aggregation, alter the rate and extent of lipid digestion, and reduce nutraceutical bioavailability.	[23,68,69]
Lipid NPs	In vitro GIT model Rat feeding study	Lipid digestion and nutraceutical bioavailability increased with decreasing droplet size. The encapsulation of vitamins in indigestible mineral oil droplets reduces their bioaccessibility.	[75,76,77]
Protein NPs	In vitro GIT model Rat feeding study	Protein nanoparticles undergo aggregation and digestion. Protein-based nano-carriers have a high encapsulation efficiency and loading capacity of bioactives.	[78,79]

## References

[1] Nile S.H., Baskar V., Selvaraj D., Nile A., Xiao J., Kai G. (2020). Nanotechnologies in Food Science: Applications, Recent Trends, and Future Perspectives. Nano-Micro Lett..

[2] Luo Y. (2020). Perspectives on important considerations in designing nanoparticles for oral delivery applications in food. J. Agric. Food Res..

[3] Shah S., Nene S., Rangaraj N., Raghuvanshi R.S., Singh S.B., Srivastava S. (2020). Bridging the gap: Academia, industry and FDA convergence for nanomaterials. Drug Dev. Ind. Pharm..

[4] Xavier M., Parente I.A., Rodrigues P.M., Cerqueira M.A., Pastrana L., Gonçalves C. (2021). Safety and fate of nanomaterials in food: The role of in vitro tests. Trends Food Sci. Technol..

[5] Prajitha N., Athira S.S., Mohanan P.V. (2019). Bio-interactions and risks of engineered nanoparticles. Environ. Res..

[6] Liu X., Zhang B., Sohal I.S., Bello D., Chen H., Lim L.-T., Rogers M. (2019). Chapter Eight—Is “nano safe to eat or not”? A review of the state-of-the art in soft engineered nanoparticle (sENP) formulation and delivery in foods. Advances in Food and Nutrition Research.

[7] Cao Y., Li J., Liu F., Li X.Y., Jiang Q., Cheng S.S., Gu Y.X. (2016). Consideration of interaction between nanoparticles and food components for the safety assessment of nanoparticles following oral exposure: A review. Environ. Toxicol. Pharmacol..

[8] McClements D.J., DeLoid G., Pyrgiotakis G., Shatkin J.A., Xiao H., Demokritou P. (2016). The role of the food matrix and gastrointestinal tract in the assessment of biological properties of ingested engineered nanomaterials (iENMs): State of the science and knowledge gaps. NanoImpact.

[9] McClements D.J., Xiao H., Demokritou P. (2017). Physicochemical and colloidal aspects of food matrix effects on gastrointestinal fate of ingested inorganic nanoparticles. Adv. Colloid Interface Sci..

[10] Moustafa H., Darwish N.A., Youssef A.M. (2022). Rational formulations of sustainable polyurethane/chitin/rosin composites reinforced with ZnO-doped-SiO2 nanoparticles for green packaging applications. Food Chem..

[11] Moustafa H., El-Sayed S.M., Youssef A.M. (2021). Synergistic impact of cumin essential oil on enhancing of UV-blocking and antibacterial activity of biodegradable poly(butylene adipate-co-terephthalate)/clay platelets nanocomposites. J. Thermoplast. Compos. Mater..

[12] Moustafa H., Karmalawi A.M., Youssef A.M. (2021). Development of dapsone-capped TiO2 hybrid nanocomposites and their effects on the UV radiation, mechanical, thermal properties and antibacterial activity of PVA bionanocomposites. Environ. Nanotechnol. Monit. Manag..

[13] McClements D.J., Xiao H. (2017). Is nano safe in foods? Establishing the factors impacting the gastrointestinal fate and toxicity of organic and inorganic food-grade nanoparticles. NPJ Sci. Food.

[14] Fröhlich E.E., Fröhlich E. (2016). Cytotoxicity of nanoparticles contained in food on intestinal cells and the gut microbiota. Int. J. Mol. Sci..

[15] Maurer-Jones M.A., Gunsolus I.L., Murphy C.J., Haynes C.L. (2013). Toxicity of Engineered Nanoparticles in the Environment. Anal. Chem..

[16] Lu W., Nishinari K., Phillips G.O., Fang Y. (2021). Colloidal nutrition science to understand food-body interaction. Trends Food Sci. Technol..

[17] Banerjee A., Qi J., Gogoi R., Wong J., Mitragotri S. (2016). Role of nanoparticle size, shape and surface chemistry in oral drug delivery. J. Control. Release.

[18] Pan K., Zhong Q. (2016). Organic Nanoparticles in Foods: Fabrication, Characterization, and Utilization. Annu. Rev. Food Sci. Technol..

[19] Sohal I.S., Cho Y.K., O’Fallon K.S., Gaines P., Demokritou P., Bello D. (2018). Dissolution behavior and biodurability of ingested engineered nanomaterials in the gastrointestinal environment. ACS Nano.

[20] Moradi M., Razavi R., Omer A.K., Farhangfar A., McClements D.J. (2022). Interactions between nanoparticle-based food additives and other food ingredients: A review of current knowledge. Trends Food Sci. Technol..

[21] Zhang R., Zhang Z., Li R., Tan Y., Lv S., McClements D.J. (2020). Impact of Pesticide Type and Emulsion Fat Content on the Bioaccessibility of Pesticides in Natural Products. Molecules.

[22] Koshani R., Madadlou A. (2018). A viewpoint on the gastrointestinal fate of cellulose nanocrystals. Trends Food Sci. Technol..

[23] Mackie A., Gourcy S., Rigby N., Moffat J., Capron I., Bajka B. (2019). The fate of cellulose nanocrystal stabilised emulsions after simulated gastrointestinal digestion and exposure to intestinal mucosa. Nanoscale.

[24] Qiu C., Wang C., Gong C., McClements D.J., Jin Z., Wang J. (2020). Advances in research on preparation, characterization, interaction with proteins, digestion and delivery systems of starch-based nanoparticles. Int. J. Biol. Macromol..

[25] Melikoğlu A.Y., Bilek S.E., Cesur S. (2019). Optimum alkaline treatment parameters for the extraction of cellulose and production of cellulose nanocrystals from apple pomace. Carbohydr. Polym..

[26] Lv S., Zhou H., Bai L., Rojas O.J., McClements D.J. (2021). Development of food-grade Pickering emulsions stabilized by a mixture of cellulose nanofibrils and nanochitin. Food Hydrocoll..

[27] Qin D., Yang X., Gao S., Yao J., McClements D.J. (2017). Influence of dietary fibers on lipid digestion: Comparison of single-stage and multiple-stage gastrointestinal models. Food Hydrocoll..

[28] Cho Y.-H., Jones O.G., Lim L.-T., Rogers M. (2019). Chapter Two—Assembled protein nanoparticles in food or nutrition applications. Advances in Food and Nutrition Research.

[29] Wang Y., Yan W., Li R., Jia X., Cheng Y. (2019). Impact of deamidation on gliadin-based nanoparticle formation and curcumin encapsulation. J. Food Eng..

[30] Zou L., Zheng B., Zhang R., Zhang Z., Liu W., Liu C., Xiao H., McClements D.J. (2016). Enhancing the bioaccessibility of hydrophobic bioactive agents using mixed colloidal dispersions: Curcumin-loaded zein nanoparticles plus digestible lipid nanoparticles. Food Res. Int..

[31] Safaya M., Rotliwala Y.C. (2020). Nanoemulsions: A review on low energy formulation methods, characterization, applications and optimization technique. Mater. Today Proc..

[32] McClements D.J. (2021). Advances in edible nanoemulsions: Digestion, bioavailability, and potential toxicity. Prog. Lipid Res..

[33] Tan Y., Zhang Z., Muriel Mundo J., McClements D.J. (2020). Factors impacting lipid digestion and nutraceutical bioaccessibility assessed by standardized gastrointestinal model (INFOGEST): Emulsifier type. Food Res. Int..

[34] Borreani J., Leonardi C., Moraga G., Quiles A., Hernando I. (2019). How do Different Types of Emulsifiers/Stabilizers Affect the In Vitro Intestinal Digestion of O/W Emulsions?. Food Biophys..

[35] Joye I.J., Davidov-Pardo G., McClements D.J. (2014). Nanotechnology for increased micronutrient bioavailability. Trends Food Sci. Technol..

[36] Burger T.G., Zhang Y. (2019). Recent progress in the utilization of pea protein as an emulsifier for food applications. Trends Food Sci. Technol..

[37] De Matteis V. (2017). Exposure to Inorganic Nanoparticles: Routes of Entry, Immune Response, Biodistribution and In Vitro/In Vivo Toxicity Evaluation. Toxics.

[38] Weir A., Westerhoff P., Fabricius L., Hristovski K., von Goetz N. (2012). Titanium dioxide nanoparticles in food and personal care products. Environ. Sci. Technol..

[39] Sanches P.L., Geaquinto L.R.d.O., Cruz R., Schuck D.C., Lorencini M., Granjeiro J.M., Ribeiro A.R.L. (2020). Toxicity Evaluation of TiO_2_ Nanoparticles on the 3D Skin Model: A Systematic Review. Front. Bioeng. Biotechnol..

[40] Baranowska-Wójcik E., Szwajgier D., Oleszczuk P., Winiarska-Mieczan A. (2020). Effects of Titanium Dioxide Nanoparticles Exposure on Human Health—A Review. Biol. Trace Elem. Res..

[41] Boutillier S., Fourmentin S., Laperche B. (2020). Food additives and the future of health: An analysis of the ongoing controversy on titanium dioxide. Futures.

[42] Kose O., Tomatis M., Leclerc L., Belblidia N.-B., Hochepied J.-F., Turci F., Pourchez J., Forest V. (2020). Impact of the Physicochemical Features of TiO2 Nanoparticles on Their In Vitro Toxicity. Chem. Res. Toxicol..

[43] Cao X., Zhang T., DeLoid G.M., Gaffrey M.J., Weitz K.K., Thrall B.D., Qian W.-J., Demokritou P. (2020). Evaluation of the cytotoxic and cellular proteome impacts of food-grade TiO_2_ (E171) using simulated gastrointestinal digestions and a tri-culture small intestinal epithelial model. NanoImpact.

[44] Chen Z., Han S., Zhou S., Feng H., Liu Y., Jia G. (2020). Review of health safety aspects of titanium dioxide nanoparticles in food application. NanoImpact.

[45] Younes M., Aggett P., Aguilar F., Crebelli R., Dusemund B., Filipič M., Frutos M.J., Galtier P., Gottet D., EFSA Panel on Food Additives and Nutrient Sources added to Food (ANS) (2018). Re-evaluation of silicon dioxide (E 551) as a food additive. EFSA J..

[46] Yang Y., Faust J.J., Schoepf J., Hristovski K., Capco D.G., Herckes P., Westerhoff P. (2016). Survey of food-grade silica dioxide nanomaterial occurrence, characterization, human gut impacts and fate across its lifecycle. Sci. Total Environ..

[47] Liman R., Acikbas Y., Ciğerci İ.H., Ali M.M., Kars M.D. (2020). Cytotoxic and Genotoxic Assessment of Silicon Dioxide Nanoparticles by Allium and Comet Tests. Bull. Environ. Contam. Toxicol..

[48] Yun J.W., Kim S.H., You J.R., Kim W.H., Jang J.J., Min S.K., Kim H.C., Chung D.H., Jeong J., Kang B.C. (2015). Comparative toxicity of silicon dioxide, silver and iron oxide nanoparticles after repeated oral administration to rats. J. Appl. Toxicol..

[49] Palanog A.D., Calayugan M.I.C., Descalsota-Empleo G.I., Amparado A., Inabangan-Asilo M.A., Arocena E.C., Sta. Cruz P.C., Borromeo T.H., Lalusin A., Hernandez J.E. (2019). Zinc and Iron Nutrition Status in the Philippines Population and Local Soils. Front. Nutr..

[50] Sohal I.S., DeLoid G.M., O’Fallon K.S., Gaines P., Demokritou P., Bello D. (2020). Effects of ingested food-grade titanium dioxide, silicon dioxide, iron (III) oxide and zinc oxide nanoparticles on an in vitro model of intestinal epithelium: Comparison between monoculture vs. a mucus-secreting coculture model. NanoImpact.

[51] Shen C., James S.A., de Jonge M.D., Turney T.W., Wright P.F.A., Feltis B.N. (2013). Relating Cytotoxicity, Zinc Ions, and Reactive Oxygen in ZnO Nanoparticle–Exposed Human Immune Cells. Toxicol. Sci..

[52] Assadian E., Dezhampanah H., Seydi E., Pourahmad J. (2019). Toxicity of Fe_2_O_3_ nanoparticles on human blood lymphocytes. J. Biochem. Mol. Toxicol..

[53] Petters C., Irrsack E., Koch M., Dringen R. (2014). Uptake and Metabolism of Iron Oxide Nanoparticles in Brain Cells. Neurochem. Res..

[54] Fulgoni III V.L., Keast D.R., Bailey R.L., Dwyer J. (2011). Foods, fortificants, and supplements: Where do Americans get their nutrients?. J. Nutr..

[55] Hilty F.M., Arnold M., Hilbe M., Teleki A., Knijnenburg J.T.N., Ehrensperger F., Hurrell R.F., Pratsinis S.E., Langhans W., Zimmermann M.B. (2010). Iron from nanocompounds containing iron and zinc is highly bioavailable in rats without tissue accumulation. Nat. Nanotechnol..

[56] Voss L., Hoché E., Stock V., Böhmert L., Braeuning A., Thünemann A.F., Sieg H. (2021). Intestinal and hepatic effects of iron oxide nanoparticles. Arch. Toxicol..

[57] Sim W., Barnard R.T., Blaskovich M.A.T., Ziora Z.M. (2018). Antimicrobial Silver in Medicinal and Consumer Applications: A Patent Review of the Past Decade (2007–2017). Antibiotics.

[58] Rónavári A., Igaz N., Adamecz D.I., Szerencsés B., Molnar C., Kónya Z., Pfeiffer I., Kiricsi M. (2021). Green Silver and Gold Nanoparticles: Biological Synthesis Approaches and Potentials for Biomedical Applications. Molecules.

[59] Elahi N., Kamali M., Baghersad M.H. (2018). Recent biomedical applications of gold nanoparticles: A review. Talanta.

[60] Dykman L., Khlebtsov N. (2012). Gold nanoparticles in biomedical applications: Recent advances and perspectives. Chem. Soc. Rev..

[61] Burdușel A.-C., Gherasim O., Grumezescu A.M., Mogoantă L., Ficai A., Andronescu E. (2018). Biomedical Applications of Silver Nanoparticles: An Up-to-Date Overview. Nanomaterials.

[62] Simbine E.O., Rodrigues L.d.C., Lapa-GuimarÃEs J., Kamimura E.S., Corassin C.H., Oliveira C.A.F.d. (2019). Application of silver nanoparticles in food packages: A review. Food Sci. Technol..

[63] Hendrickson O.D., Klochkov S.G., Novikova O.V., Bravova I.M., Shevtsova E.F., Safenkova I.V., Zherdev A.V., Bachurin S.O., Dzantiev B.B. (2016). Toxicity of nanosilver in intragastric studies: Biodistribution and metabolic effects. Toxicol. Lett..

[64] Sundaresan V., Monaghan J.W., Willets K.A. (2018). Visualizing the Effect of Partial Oxide Formation on Single Silver Nanoparticle Electrodissolution. J. Phys. Chem. C.

[65] Pinďáková L., Kašpárková V., Kejlová K., Dvořáková M., Krsek D., Jírová D., Kašparová L. (2017). Behaviour of silver nanoparticles in simulated saliva and gastrointestinal fluids. Int. J. Pharm..

[66] König J., Wells J., Cani P.D., García-Ródenas C.L., MacDonald T., Mercenier A., Whyte J., Troost F., Brummer R.-J. (2016). Human Intestinal Barrier Function in Health and Disease. Clin. Transl. Gastroenterol..

[67] Brodkorb A., Egger L., Alminger M., Alvito P., Assunção R., Ballance S., Bohn T., Bourlieu-Lacanal C., Boutrou R., Carrière F. (2019). INFOGEST static in vitro simulation of gastrointestinal food digestion. Nat. Protoc..

[68] Zhou H., Pandya J.K., Tan Y., Liu J., Peng S., Muriel Mundo J.L., He L., Xiao H., McClements D.J. (2019). Role of Mucin in Behavior of Food-Grade TiO_2_ Nanoparticles under Simulated Oral Conditions. J. Agric. Food Chem..

[69] Zhou H., Tan Y., Lv S., Liu J., Muriel Mundo J.L., Bai L., Rojas O.J., McClements D.J. (2020). Nanochitin-stabilized pickering emulsions: Influence of nanochitin on lipid digestibility and vitamin bioaccessibility. Food Hydrocoll..

[70] Li C., Yu W., Wu P., Chen X.D. (2020). Current in vitro digestion systems for understanding food digestion in human upper gastrointestinal tract. Trends Food Sci. Technol..

[71] Mosele J.I., Macià A., Romero M.-P., Motilva M.-J., Rubió L. (2015). Application of in vitro gastrointestinal digestion and colonic fermentation models to pomegranate products (juice, pulp and peel extract) to study the stability and catabolism of phenolic compounds. J. Funct. Foods.

[72] Coreas R., Cao X., DeLoid G.M., Demokritou P., Zhong W. (2020). Lipid and protein corona of food-grade TiO_2_ nanoparticles in simulated gastrointestinal digestion. NanoImpact.

[73] Li Q., Li T., Liu C., DeLoid G., Pyrgiotakis G., Demokritou P., Zhang R., Xiao H., McClements D.J. (2017). Potential impact of inorganic nanoparticles on macronutrient digestion: Titanium dioxide nanoparticles slightly reduce lipid digestion under simulated gastrointestinal conditions. Nanotoxicology.

[74] Laloux L., Kastrati D., Cambier S., Gutleb A.C., Schneider Y.-J. (2020). The Food Matrix and the Gastrointestinal Fluids Alter the Features of Silver Nanoparticles. Small.

[75] Tan Y., Zhang Z., Liu J., Xiao H., McClements D.J. (2020). Factors impacting lipid digestion and nutraceutical bioaccessibility assessed by standardized gastrointestinal model (INFOGEST): Oil droplet size. Food Funct..

[76] Tan Y., Liu J., Zhou H., Muriel Mundo J., McClements D.J. (2019). Impact of an indigestible oil phase (mineral oil) on the bioaccessibility of vitamin D3 encapsulated in whey protein-stabilized nanoemulsions. Food Res. Int..

[77] Infantes-Garcia M.R., Verkempinck S.H.E., Guevara-Zambrano J.M., Andreoletti C., Hendrickx M.E., Grauwet T. (2020). Enzymatic and chemical conversions taking place during in vitro gastric lipid digestion: The effect of emulsion droplet size behavior. Food Chem..

[78] Liu Q., Cheng J., Sun X., Guo M. (2021). Preparation, characterization, and antioxidant activity of zein nanoparticles stabilized by whey protein nanofibrils. Int. J. Biol. Macromol..

[79] Tang C.-H. (2021). Assembly of food proteins for nano- encapsulation and delivery of nutraceuticals (a mini-review). Food Hydrocoll..

[80] Wang Y., Li M., Xu X., Tang W., Xiong L., Sun Q. (2019). Formation of Protein Corona on Nanoparticles with Digestive Enzymes in Simulated Gastrointestinal Fluids. J. Agric. Food Chem..

[81] Wang Y., Sun Y., Li M., Xiong L., Xu X., Ji N., Dai L., Sun Q. (2020). The formation of a protein corona and the interaction with α-amylase by chitin nanowhiskers in simulated saliva fluid. Food Hydrocoll..

[82] Peng Q., Liu J., Zhang T., Zhang T.-X., Zhang C.-L., Mu H. (2019). Digestive Enzyme Corona Formed in the Gastrointestinal Tract and Its Impact on Epithelial Cell Uptake of Nanoparticles. Biomacromolecules.

[83] Stebounova L.V., Guio E., Grassian V.H. (2011). Silver nanoparticles in simulated biological media: A study of aggregation, sedimentation, and dissolution. J. Nanoparticle Res..

[84] Ault A.P., Stark D.I., Axson J.L., Keeney J.N., Maynard A.D., Bergin I.L., Philbert M.A. (2016). Protein corona-induced modification of silver nanoparticle aggregation in simulated gastric fluid. Environ. Sci. Nano.

[85] Lozsan A. (2012). Salt-induced fast aggregation of nano-emulsions: Structural and kinetic scaling. Colloid Polym. Sci..

[86] Liang L., Zhang X., Wang X., Jin Q., McClements D.J. (2018). Influence of Dairy Emulsifier Type and Lipid Droplet Size on Gastrointestinal Fate of Model Emulsions: In Vitro Digestion Study. J. Agric. Food Chem..

[87] Zhang R., Wu W., Zhang Z., Lv S., Xing B., McClements D.J. (2019). Impact of Food Emulsions on the Bioaccessibility of Hydrophobic Pesticide Residues in Co-Ingested Natural Products: Influence of Emulsifier and Dietary Fiber Type. J. Agric. Food Chem..

[88] Zaeim D., Mulet-Cabero A.-I., Read S.A., Liu W., Han J., Wilde P.J. (2022). Effect of oil droplet size on the gastric digestion of milk protein emulsions using a semi-dynamic gastric model. Food Hydrocoll..

[89] Zhai H.L., Gunness P., Gidley M.J. (2020). Barley beta-glucan effects on emulsification and in vitro lipolysis of canola oil are modulated by molecular size, mixing method, and emulsifier type. Food Hydrocoll..

[90] Espinal-Ruiz M., Parada-Alfonso F., Restrepo-Sánchez L.-P., Narváez-Cuenca C.-E., McClements D.J. (2014). Impact of dietary fibers [methyl cellulose, chitosan, and pectin] on digestion of lipids under simulated gastrointestinal conditions. Food Funct..

[91] Zhou H., Dai T., Liu J., Tan Y., Bai L., Rojas O.J., McClements D.J. (2021). Chitin nanocrystals reduce lipid digestion and β-carotene bioaccessibility: An in-vitro INFOGEST gastrointestinal study. Food Hydrocoll..

[92] Kong H., Wu F., Jiang X., Wang T., Hu M., Chen J., Huang W., Bao Y., Wang Y. (2019). Nano-TiO_2_ impairs digestive enzyme activities of marine mussels under ocean acidification. Chemosphere.

[93] DeLoid G.M., Sohal I.S., Lorente L.R., Molina R.M., Pyrgiotakis G., Stevanovic A., Zhang R., McClements D.J., Geitner N.K., Bousfield D.W. (2018). Reducing Intestinal Digestion and Absorption of Fat Using a Nature-Derived Biopolymer: Interference of Triglyceride Hydrolysis by Nanocellulose. ACS Nano.

[94] Sadiq U., Gill H., Chandrapala J. (2021). Casein Micelles as an Emerging Delivery System for Bioactive Food Components. Foods.

[95] Cao X., Han Y., Li F., Li Z., McClements D.J., He L., Decker E.A., Xing B., Xiao H. (2019). Impact of protein-nanoparticle interactions on gastrointestinal fate of ingested nanoparticles: Not just simple protein corona effects. NanoImpact.

[96] Nile S.H., Park S.W. (2014). Edible berries: Bioactive components and their effect on human health. Nutrition.

[97] Recharla N., Riaz M., Ko S., Park S. (2017). Novel technologies to enhance solubility of food-derived bioactive compounds: A review. J. Funct. Foods.

[98] Jafari S.M., McClements D.J., Toldrá F. (2017). Chapter One—Nanotechnology Approaches for Increasing Nutrient Bioavailability. Advances in Food and Nutrition Research.

[99] Yao M., Li Z., McClements D.J., Tang Z., Xiao H. (2020). Design of nanoemulsion-based delivery systems to enhance intestinal lymphatic transport of lipophilic food bioactives: Influence of oil type. Food Chem..

[100] Yao M., McClements D.J., Xiao H. (2015). Improving oral bioavailability of nutraceuticals by engineered nanoparticle-based delivery systems. Curr. Opin. Food Sci..

[101] Zhan X., Dai L., Zhang L., Gao Y. (2020). Entrapment of curcumin in whey protein isolate and zein composite nanoparticles using pH-driven method. Food Hydrocoll..

[102] Wu W., Li T., Zheng Y. (2021). Editorial of Special Issue “The Biological Fate of Drug Nanocarriers”. Acta Pharm. Sin. B.

[103] Xu Y., Michalowski C.B., Beloqui A. (2021). Advances in lipid carriers for drug delivery to the gastrointestinal tract. Curr. Opin. Colloid Interface Sci..

[104] Salvia-Trujillo L., Qian C., Martín-Belloso O., McClements D.J. (2013). Influence of particle size on lipid digestion and β-carotene bioaccessibility in emulsions and nanoemulsions. Food Chem..

[105] Cho H.T., Salvia-Trujillo L., Kim J., Park Y., Xiao H., McClements D.J. (2014). Droplet size and composition of nutraceutical nanoemulsions influences bioavailability of long chain fatty acids and Coenzyme Q10. Food Chem..

[106] Speranza A., Corradini M.G., Hartman T.G., Ribnicky D., Oren A., Rogers M.A. (2013). Influence of Emulsifier Structure on Lipid Bioaccessibility in Oil–Water Nanoemulsions. J. Agric. Food Chem..

[107] Tan Y., Li R., Zhou H., Liu J., Mundo J.L.M., Zhang R., McClements D.J. (2020). Impact of calcium levels on lipid digestion and nutraceutical bioaccessibility in nanoemulsion delivery systems studied using standardized INFOGEST digestion protocol. Food Funct..

[108] Zhou H., Zheng B., Zhang Z., Zhang R., He L., McClements D.J. (2021). Fortification of Plant-Based Milk with Calcium May Reduce Vitamin D Bioaccessibility: An In Vitro Digestion Study. J. Agric. Food Chem..

